# Baseline retinal nerve fiber layer thickness as a predictive biomarker for endoscopic optic canal decompression in NAION: towards a precision medicine approach

**DOI:** 10.1186/s12967-026-08361-1

**Published:** 2026-06-08

**Authors:** Zhiwen Yao, Ziwen Chen, Bo Yu, Nanxin Wu, Ruirui Hu, Haotian Peng, Miyao Zhu, Wencan Wu, Wentao Yan

**Affiliations:** 1 Eye Research Center, Hangzhou Institute of Medicine, Chinese Academy of Sciences, Wenzhou Medical University Eye Hospital, Hangzhou, Zhejiang China; 2 Oujiang Laboratory (Zhejiang Lab for Regenerative Medicine, Vision and Brain Health), Wenzhou, Zhejiang 325000 China; 3 Zhejiang Key Laboratory of Key Technologies for Visual Pathway Reconstruction, Department of Orbital and Oculoplastic Surgery, Wenzhou Medical University Eye Hospital, Wenzhou, China

**Keywords:** Ischemic optic neuropathy, Decompression surgery, Endoscopy

## Abstract

**Background:**

Nonarteritic anterior ischemic optic neuropathy (NAION) remains a major cause of blindness with no consensus on treatment. The heterogeneity of patient outcomes suggests that a “one-size-fits-all” approach is ineffective. This study aimed to identify structural biomarkers to define the therapeutic window for endoscopic transnasal optic canal decompression (ETOCD) and explore its mechanism via vascular reperfusion imaging.

**Methods:**

Seventy-one patients diagnosed with NAION were included and categorized into two groups: an ETOCD group (*n* = 30) and a medical management group (*n* = 41). Best-corrected visual acuity (BCVA), visual field index (VFI), mean deviation (MD), pattern standard deviation (PSD), and retinal nerve fiber layer (RNFL) thickness were assessed at baseline and 3 months after treatment. Optical coherence tomography angiography (OCTA) was utilized to evaluate microvascular recovery. Multivariable regression and interaction analyses were performed to investigate RNFL as a predictive biomarker.

**Results:**

While ETOCD showed superior overall efficacy (Adjusted β = -0.41, *P* < 0.001), a critical treatment-by-biomarker interaction was identified (P for interaction = 0.014). Patients with moderate edema (RNFL < 150 μm) exhibited a profound therapeutic response (OR 7.88, 95% CI: 2.07–29.94, *P* = 0.002), whereas those with massive edema derived minimal benefit. OCTA analysis in responders revealed significant radial peripapillary capillary reperfusion, providing mechanistic support of the “osseous compartment syndrome” hypothesis.

**Conclusion:**

We identified baseline RNFL thickness (< 150 μm) as a potential predictive biomarker for surgical success in NAION. These findings support an alternative therapeutic strategy from empiric treatment to biomarker-guided precision decompression, bridging the gap between anatomical pathology and surgical intervention.

**Trial registration:**

Retrospectively registered.

## Key message

**Unmet need**: NAION lacks effective therapies, and patient selection for surgery remains controversial.

**Biomarker discovery**: We identified baseline RNFL thickness (< 150 μm) as a potential predictive biomarker for surgical response.

**Mechanism**: OCTA imaging suggests that ETOCD facilitates microvascular reperfusion by relieving the osseous compartment syndrome.

**Impact**: This study proposes a precision medicine approach, using structural biomarkers to guide surgical decision-making in NAION.

## Background

Nonarteritic anterior ischemic optic neuropathy (NAION) is one of the most common acute optic neuropathies in adults over 50 years of age, characterized by sudden, painless, and often permanent unilateral vision loss, accompanied by optic disc edema and altitudinal visual field defects [1, 2]. Despite its considerable clinical burden, NAION remains a condition with no consistently effective treatment [3, 4].

The pathophysiology of NAION is attributed to impaired perfusion of the short posterior ciliary arteries supplying the optic nerve head [5]. A proposed mechanism is that the initial ischemic insult at the level of the lamina cribrosa leads to axonal edema, which may then extend posteriorly into the rigid bony optic canal [6]. This propagation could result in a secondary “osseous compartment syndrome,” compromising venous outflow and exacerbating the primary ischemic injury—a mechanism that may explain the rapid vision loss in the early phase of the disease [7].

Current management strategies for NAION are primarily supportive, focusing on systemic vascular risk factor modification (e.g., hypertension, diabetes, dyslipidemia) to reduce the risk of fellow-eye involvement [8, 9]. A range of pharmacologic interventions—including systemic corticosteroids, neuroprotective agents, and intravitreal anti-VEGF or steroid injections—have been investigated, yet none has demonstrated consistent efficacy in restoring or significantly improving vision in the affected eye [3, 10–12].

The concept of surgical decompression for NAION is rooted in relieving mechanical constraint within the optic canal. Notably, the Ischemic Optic Neuropathy Decompression Trial (IONDT) found optic nerve sheath fenestration (ONSF) ineffective [13]; however, ONSF targets dural pressure rather than the bony canal itself. In contrast, endoscopic transnasal optic canal decompression (ETOCD) directly addresses the osseous confines of the canal, potentially alleviating compartment physiology and improving local perfusion [14, 15]. Advances in endoscopic and image-guided techniques have enhanced the safety and precision of this approach, supporting its study in compressive and traumatic optic neuropathies [16, 17].

Against this background, the present study was designed to evaluate whether ETOCD can improve visual outcomes in NAION compared with medical management alone. We further aimed to identify structural biomarkers—in particular, baseline retinal nerve fiber layer (RNFL) thickness—that may predict therapeutic response. By investigating both functional and anatomical outcomes, this work seeks to address a significant unmet need in neuro-ophthalmology and to inform future targeted intervention strategies. Specifically, we aimed to define the ‘structural therapeutic window’ where decompression can rescue viable axons before irreversible infarction occurs, bridging the gap between anatomical pathology and surgical intervention.

## Methods

### Study design and population

This single-center retrospective cohort study included patients diagnosed with NAION at the Eye Hospital of Wenzhou Medical University between March 2021 and November 2023. The study protocol was approved by the institutional ethics committee, and the requirement for informed consent was waived due to the retrospective design (Approval No.2026-030-K-022). All patient data were extracted from electronic medical records and fully anonymized prior to analysis.

The diagnosis of NAION was based on the following criteria: (1) acute, painless loss of visual acuity and/or visual field; (2) visual field testing showing a nerve fiber bundle defect (e.g., altitudinal or quadrantic) contiguous with the blind spot; (3) optic disc edema, focal or diffuse, often with peripapillary splinter hemorrhages; and (4) relative afferent pupillary defect in unilateral cases [2].

Patients were excluded if they had a history of ocular surgery, glaucoma, or any other ocular pathology that could explain visual loss. Given the retrospective nature of this study, the diagnosis of NAION was primarily based on typical clinical presentations (e.g., sudden, painless vision loss with concurrent optic disc edema) and the absence of systemic symptoms suggestive of giant cell arteritis. Patients with atypical presentations who underwent further neuroimaging or laboratory testing and were subsequently diagnosed with compressive optic neuropathy, optic neuritis, or arteritic AION were excluded. Furthermore, patients whose clinical course during follow-up was inconsistent with NAION were also excluded. A minimum follow-up of 3 months after treatment initiation was required for inclusion.

### Treatment allocation

In this observational study, treatment assignment was non-randomized. The choice between ETOCD and medical management followed a shared decision-making process. All patients were offered standard medical management according to prevailing guidelines. ETOCD was presented as an optional intervention, particularly to those with severe visual loss at presentation (BCVA ≥ 1.0 logMAR) or without functional improvement after an initial period of observation. Comprehensive counseling included discussion of surgical risks (e.g., cerebrospinal fluid leak, vascular injury) and the absence of guaranteed efficacy. Final treatment allocation was determined by patient preference: individuals opting for surgery comprised the ETOCD group, whereas those selecting conservative care formed the medical management group. To evaluate potential selection bias, baseline demographics and clinical characteristics were compared between the two cohorts.

### Interventions

All surgical procedures were performed unilaterally under general anesthesia by a single senior orbital surgeon (Dr. WW), assisted by a dedicated neuroendoscopy team. The ETOCD procedure was performed in a standardized two-step manner to ensure comprehensive decompression. First, the medial wall of the optic canal was removed using a diamond burr to achieve bony decompression, transforming the rigid canal into an open space. Second, sheath fenestration was performed via multiple punctate incisions to directly release the intrinsic tension of the edematous nerve.

Patients assigned to the medical management group received standard conservative care aligned with current regional guidelines. The regimen emphasized systemic vascular risk factor control, including management of hypertension, hyperglycemia, and dyslipidemia. At the treating physician’s discretion, a short-term course of systemic corticosteroids (e.g., oral prednisone) or neuroprotective agents such as mouse nerve growth factor (mNGF, administered per local clinical protocols) could be administered during the acute phase.

Both groups underwent identical ophthalmic evaluation protocols at predefined follow-up intervals, as detailed in the Study Design section. Additionally, serial OCT Angiography (OCTA) imaging was performed to qualitatively assess the reperfusion of the radial peripapillary capillaries (RPC), serving as an in vivo illustration of the decompression mechanism.

### Statistical analysis

All statistical analyses were conducted using R software (version 4.2.2) and MSTATA software(www.mstata.com). Best-corrected visual acuity (BCVA) was converted to the logarithm of the minimum angle of resolution (logMAR) for all quantitative analyses. Continuous variables were summarized as mean ± standard deviation for normally distributed data or as median (interquartile range) for non-normally distributed data. Categorical variables were presented as frequencies and percentages.

Between-group comparisons of baseline characteristics and postoperative outcomes were performed using independent samples t-tests or Mann–Whitney U tests for continuous variables, and chi-square or Fisher’s exact tests for categorical variables, as appropriate. Changes in ocular parameters within each group from baseline to follow-up were assessed using paired t-tests or Wilcoxon signed-rank tests.

Univariable linear regression was first employed to evaluate crude associations between treatment assignment and visual outcomes. Subsequently, multivariable linear regression models were constructed to adjust for potential confounders, including age, baseline BCVA, and time from symptom onset to treatment. To optimize model selection, a backward stepwise approach based on the Akaike Information Criterion (AIC) was employed using the stepAIC function. Patients with missing specific clinical data points (e.g., unavailable baseline RNFL thickness in one patient) were excluded only from the specific quantitative analyses requiring those variables, utilizing an available-case analysis approach. Due to the exploratory nature of this study, P values were not adjusted for multiple comparisons; instead, findings were interpreted based on their clinical and biological consistency across multiple functional and structural metrics. All tests were two-sided, and a *P* < 0.05 was considered statistically significant.

## Results

A total of 71 patients diagnosed with NAION were included in this study: 41 patients were managed medically, and 30 underwent ETOCD. The baseline demographic and clinical characteristics of both groups are summarized in Table 1. The overall mean age was 54 ± 11 years. No significant differences were observed in age (56 ± 11 vs. 51 ± 11 years, *P* = 0.063), sex distribution (53.7% vs. 43.3% female, *P* = 0.390), laterality of involvement, or median time from symptom onset to treatment initiation (60 days [range: 15–90 days] in the medical group and 45 days [range: 30–120 days] in the ETOCD group; *P* = 0.391). The prevalence of systemic comorbidities was comparable between the two groups.

**Table 1:** Baseline demographic and clinical characteristics of the study population

Characteristic	Overall *N* = 71	Medical *N* = 41	ETOCD *N* = 30	*P* value^†^
**Age(years)**,** Mean ± SD**	54.0 ± 11.0	56.0 ± 11.0	51.0 ± 11.0	0.063
**Sex**,** n (%)**				0.390
Female	35 (49.3%)	22 (53.7%)	13 (43.3%)	
Male	36 (50.7%)	19 (46.3%)	17 (56.7%)	
**Laterality**,** n (%)**				0.705
OD (Right Eye)	36 (50.7%)	20 (48.8%)	16 (53.3%)	
OS (Left Eye)	35 (49.3%)	21 (51.2%)	14 (46.7%)	
**Time to Treatment (days)**,** Median (IQR)**	60 (20–120)	60 (15–90)	45 (30–120)	0.391
**Comorbidities**,** n (%)**				
Hypertension	23 (32.4%)	11 (26.8%)	12 (40.0%)	0.241
Diabetes Mellitus	13 (18.3%)	8 (19.5%)	5 (16.7%)	0.759
Cardiovascular Disease	2 (2.9%)	1 (2.5%)	1 (3.3%)	> 0.999

ETOCD: Endoscopic Transnasal Optic Canal Decompression

^†^P values calculated using t-test for continuous variables and Chi-square/Fisher’s exact test for categorical variables. IQR: Interquartile Range

Changes in key ocular parameters are detailed in Table 2. Functionally, the ETOCD group achieved significant gains in BCVA (*P* = 0.002) and visual field metrics (VFI *P* = 0.028; MD *P* = 0.002), markedly outperforming the medical group where vision remained static or worsened (between-group BCVA difference *P* < 0.001). Structurally, while progressive RNFL thinning occurred in both groups (reflecting expected atrophy), the ETOCD group showed significantly less thinning in the nasal quadrant (*P* = 0.034). Regarding microvascular assessment, post-operative OCTA (available in the surgical cohort) was evaluated qualitatively. Representative responders (e.g., Case 2) demonstrated visual resurgence of peripapillary capillaries, providing qualitative support for the observed structural benefits.

**Table 2:** Comparison of changes in visual and structural outcomes between treatment groups from baseline to 3 months

Characteristic	Medical (*N* = 41)		ETOCD (*N* = 30)		*P* Value for Change ‡
	Baseline	3 Months	Baseline	3 Months	
**BCVA (logMAR)**	0.80 (0.20,1.60)	1.20 (0.40,1.70)	1.20 (0.80,1.80)	0.80 (0.40, 1.30)	< 0.001
*Within-group P†*		*0.050*		*0.002*	
**VFI(%)**	67 (33, 87)	60 (36, 81)	16 (4, 46)	33 (14, 51)	0.029
*Within-group P†*		*0.989*		*0.028*	
**MD(dB)**	-16.0 ± 9.0	-16.0 ± 8.0	-27.0 (-31, -21)	-25.0 ± 7.0	0.130
*Within-group P†*		*0.512*		*0.002*	
**RNFL Thickness (µm)**					
Average	113 (71, 211)	77 (55, 101)	84 (56, 120)	63 (53, 89)	0.133
Nasal Quadrant	94 (59, 150)	60 (42, 76)	69 (48, 86)	50 (41, 71)	0.034

ETOCD: Endoscopic Transnasal Optic Canal Decompression; BCVA: Best Corrected Visual Acuity; VFI: Visual Field Index; MD: Mean Deviation; RNFL: Retinal Nerve Fiber Layer. † Within-group P: Calculated using the Paired Wilcoxon signed-rank test comparing baseline vs. 3-month values within each group.‡ P Value for Change: Calculated using the Mann-Whitney U test comparing the magnitude of change from baseline (Month 3 value minus Baseline value) between the Medical and ETOCD groups

**Fig. 1:**
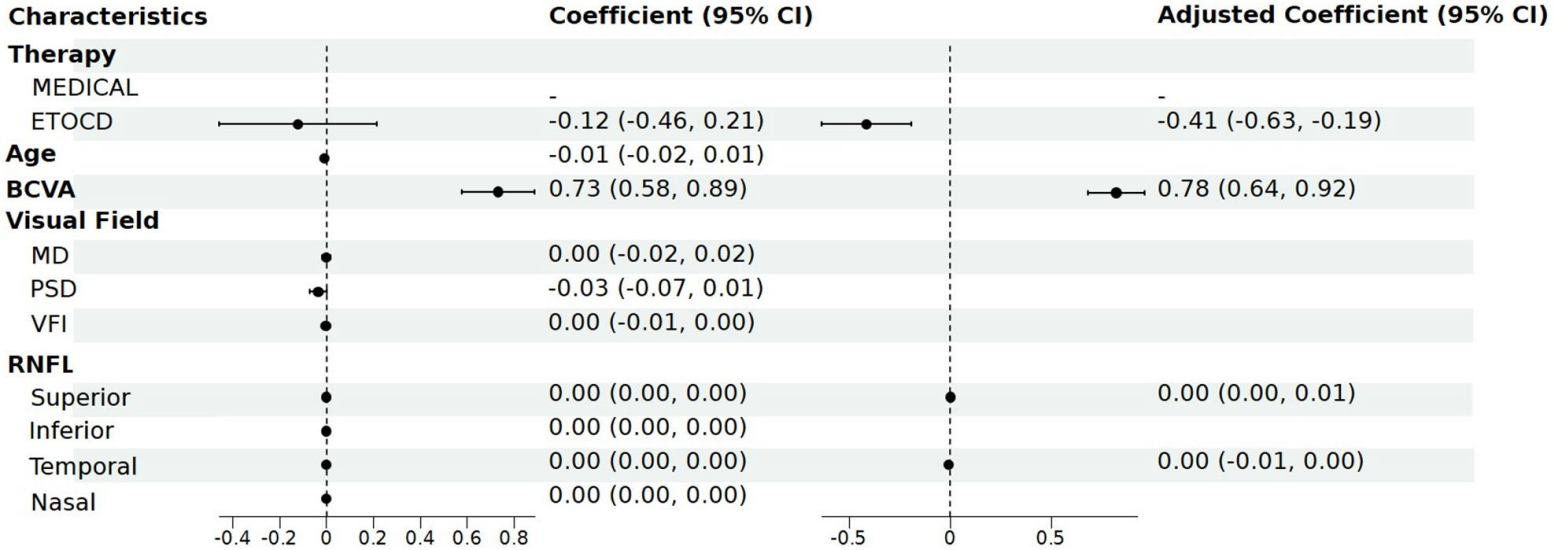
Forest plot of linear regression models for postoperative visual acuity (BCVA). Left Panel: Univariable analysis showing no statistically significant association between ETOCD and visual outcome (*P* = 0.483), likely masked by baseline severity differences. Right Panel: Multivariable analysis, adjusted for age, baseline BCVA, and time to treatment, identifies ETOCD as a significant independent predictor of visual improvement (Adjusted Coefficient − 0.41, 95% CI -0.63 to -0.19, *P* < 0.001). Error bars represent 95% confidence intervals

As illustrated in Fig. 1 and detailed in Table 3, univariable analysis initially masked the surgical benefit due to the ETOCD group’s worse baseline severity. However, after adjusting for confounders (age, time to treatment, and baseline BCVA) in the multivariable model, ETOCD emerged as a potential, independent predictor of visual recovery (Adjusted β = -0.41, 95% CI: -0.63 to -0.19; *P* < 0.001). Notably, disease duration (time from onset) showed no significant interaction with treatment outcome (*P* = 0.940), suggesting surgical efficacy persists into the subacute phase.

**Table 3:** Univariable and multivariable linear regression analyses identifying predictors of postoperative visual acuity (BCVA)

Characteristic	Univariable				Multivariable			
	*N*	Beta	95% CI	*P* value^1^	*N*	Beta	95% CI	*P* value^1^
**Therapy**								
MEDICAL	41	—	—		40	—	—	
ETOCD	30	-0.12	-0.46, 0.21	0.483	30	-0.41	-0.63, -0.19	< 0.001
**Age**	71	-0.01	-0.02, 0.01	0.282				
**Time to Treatment**	71	0.00	0.00, 0.00	0.940				
**BCVA**	71	0.73	0.58, 0.89	< 0.001	70	0.78	0.64, 0.92	< 0.001
**Visual Field**								
MD	71	0.00	-0.02, 0.02	0.917				
PSD	71	-0.03	-0.07, 0.01	0.103				
VFI	71	0.00	-0.01, 0.00	0.907				
**RNFL**								
Superior	70	0.00	0.00, 0.00	0.244	70	0.00	0.00, 0.01	0.002
Inferior	70	0.00	0.00, 0.00	0.863				
Temporal	70	0.00	0.00, 0.00	0.910	70	0.00	-0.01, 0.00	0.002
Nasal	70	0.00	0.00, 0.00	0.817				

Dependent variable: Post-treatment BCVA (logMAR). Negative β indicates improvement in visual acuity. BCVA: Best Corrected Visual Acuity; ETOCD: Endoscopic Transnasal Optic Canal Decompression; MD: Mean Deviation; PSD: Pattern Standard Deviation; VFI: Visual Field Index; RNFL: Retinal Nerve Fiber Layer. Note: The sample size for the multivariable regression model and RNFL-related analyses is *N* = 70 (rather than the total cohort of *N* = 71). Data for one patient was excluded from these specific analyses because their baseline RNFL thickness data was unavailable

**Fig. 2:**
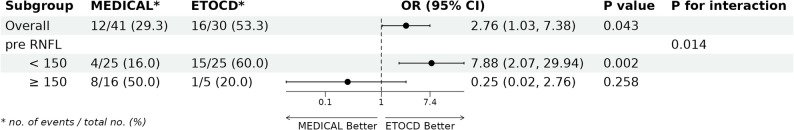
Subgroup analysis of visual improvement rates stratified by baseline RNFL thickness. A significant interaction is observed (P interaction = 0.014). Patients with moderate edema (RNFL < 150 μm) show a profound surgical benefit (OR 7.88, P = 0.002), while those with massive edema (RNFL ≥ 150 μm) show no significant benefit

Subgroup analysis based on baseline RNFL thickness revealed a significant treatment-by-subgroup interaction (P for interaction = 0.014, Table 4). The benefit of ETOCD was pronounced in patients with moderate optic disc edema (baseline RNFL < 150 μm), with an odds ratio of 7.88 (95% CI: 2.07–29.94, *P* = 0.002). In contrast, no statistically significant treatment benefit was observed in patients with massive edema (RNFL ≥ 150 μm). These results are graphically presented in the forest plot shown in Fig. 2.

**Table 4:** Subgroup analysis of visual improvement rates stratified by baseline RNFL thickness

Subgroup	MEDICAL^1^	ETOCD^1^	Crude OR (95% CI)	*P* value	*P* for interaction
Overall	12/41 (29.3)	16/30 (53.3)	2.76 (1.03–7.38)	0.043	
RNFL (µm)					0.014
< 150	4/25 (16.0)	15/25 (60.0)	7.88 (2.07–29.94)	0.002	
≥ 150	8/16 (50.0)	1/5 (20.0)	0.25 (0.02–2.76)	0.258	

Visual Improvement defined as a reduction in BCVA of 0.1 logMAR (equivalent to 1 line on an ETDRS chart) from baseline to 3 months. OR > 1 indicates higher odds of visual improvement in the ETOCD group. ETOCD: Endoscopic Transnasal Optic Canal Decompression. RNFL: Retinal Nerve Fiber Layer

### Representative case presentations

**Fig. 3:**
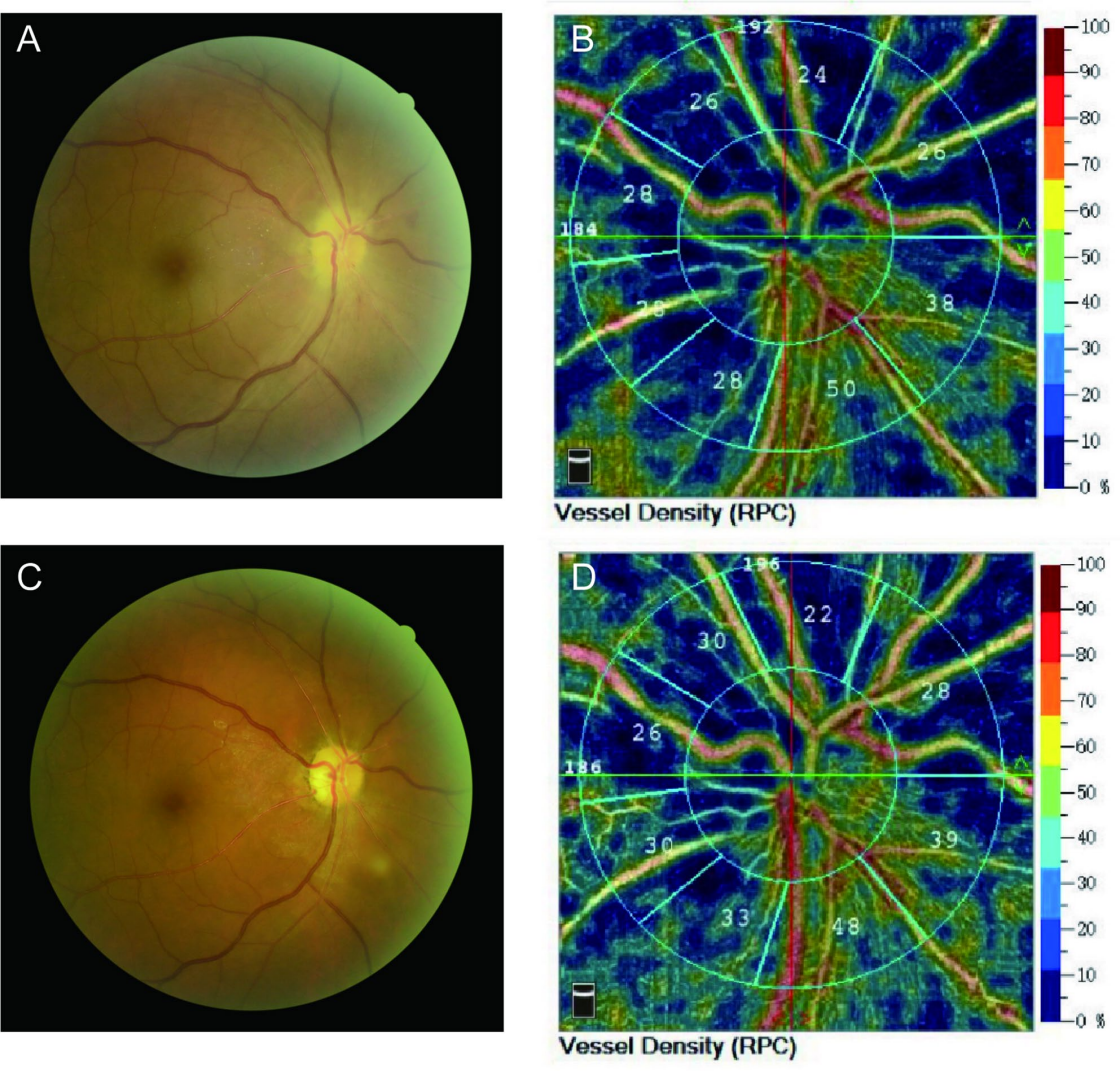
Representative Case 1: Visual illustration of structural and vascular changes in a patient with moderate edema. (**A**) Baseline fundus photography showing moderate diffuse optic disc edema. (**B**) Baseline OCT angiography (OCTA) demonstrating peripapillary capillary dropout. (**C**) Postoperative fundus photography at 3 months showing complete resolution of edema. (**D**) Postoperative OCTA at 3 months revealing improved vessel density in the radial peripapillary capillary network

Case 1 Rapid Recovery in Moderate Edema (Corresponding to Fig. 3) A 61-year-old male presented with sudden painless vision loss in the right eye (BCVA 0.7 logMAR) of 30 days’ duration. Baseline OCT revealed moderate optic disc edema (Fig. 3A) with an average RNFL thickness of 124 μm (< 150 μm threshold), and OCTA demonstrated capillary dropout in the peripapillary region (Fig. 3B). Following ETOCD, the patient reported subjective visual improvement within 24 h. At the 3-month follow-up, BCVA improved significantly to 0.4 logMAR. Structural imaging showed rapid resolution of disc edema (Fig. 3C) with preservation of the radial peripapillary capillary network (Fig. 3B vs. 3D) as observed in the overall cohort.

**Fig. 4:**
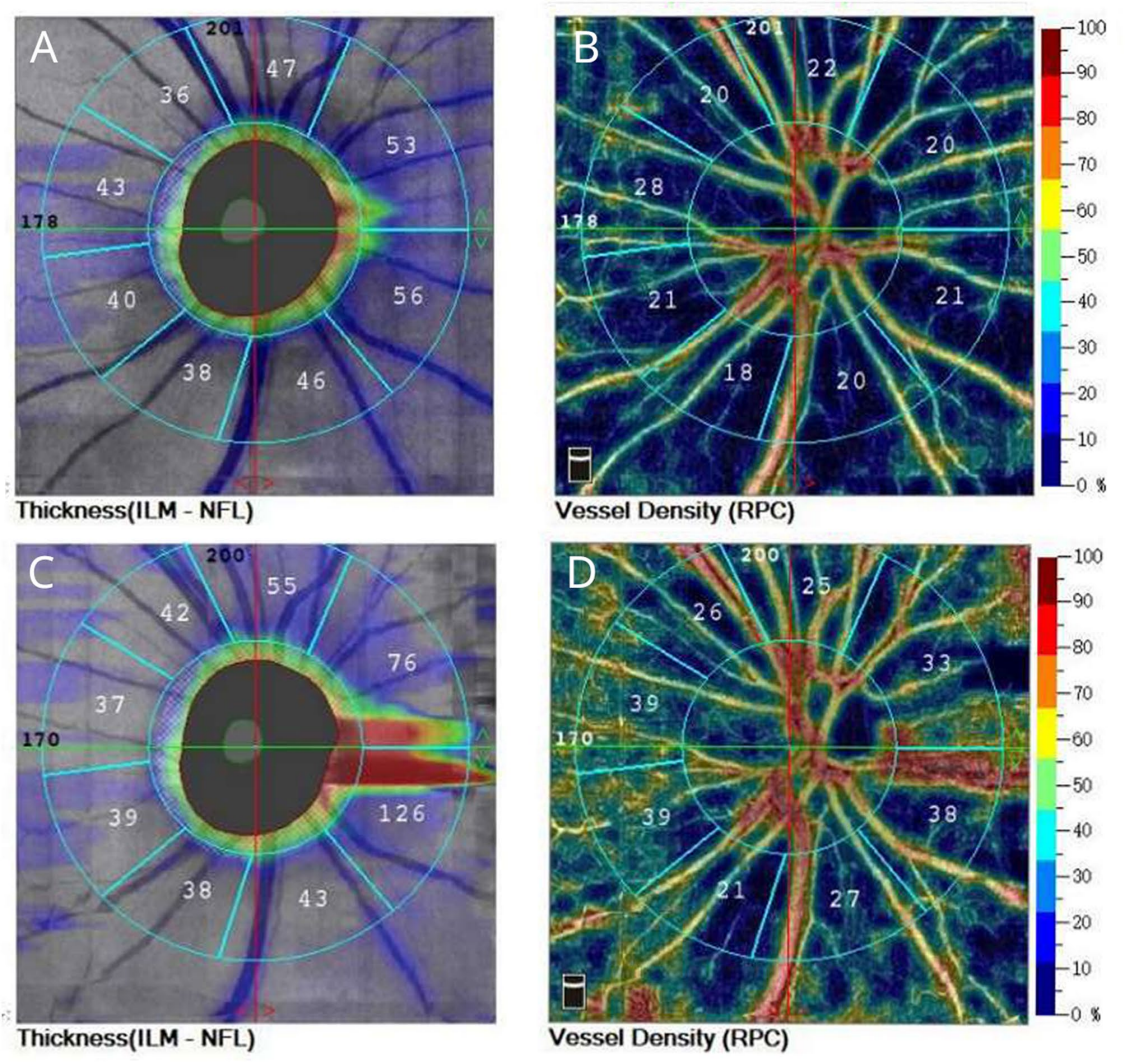
Representative Case 2: Visual illustration of structural parameters and capillary network in a patient with severe disease. (**A**, **C**) RNFL thickness maps at baseline (**A**) and 3 months postoperatively (**C**), demonstrating preservation of retinal nerve fiber layer thickness despite resolution of edema. (**B**, **D**) OCTA vessel density maps at baseline (**B**) and 3 months postoperatively (**D**), providing a visual comparison of the radial peripapillary capillaries

Case 2 Microvascular Preservation in Severe Disease (Corresponding to Fig. 4) A 62-year-old female presented with severe visual decline (BCVA 2.0 logMAR) and thinned retinal nerve fiber layer (Fig. 4A). The patient underwent ETOCD 3 months after symptom onset. Postoperative OCT at 3 months demonstrated complete resolution of the edema with a preservation of RNFL thickness (Fig. 4C). Notably, OCTA revealed a resurgence of vessel density in the radial peripapillary capillary plexus (Fig. 4B vs. 4D), suggesting that relief of the osseous compartment syndrome facilitated reperfusion of the ischemic penumbra. Her final BCVA improved to 0.7 logMAR, accompanied by an expansion of the visual field index.

## Discussion

This study suggests that ETOCD may represent a more effective therapeutic strategy than conservative medical management for selected patients with NAION.

Our principal finding is that ETOCD was associated with significant improvements in both functional and structural visual outcomes. Patients undergoing surgery demonstrated marked gains in BCVA, whereas the medically managed group showed no significant improvement and even a trend toward deterioration. This functional benefit was further supported by significant recovery in visual field indices (VFI and MD) observed exclusively in the surgical cohort. Given that NAION remains a leading cause of acute vision loss in older adults with no universally accepted therapy, these findings position ETOCD as a potentially viable intervention for altering the disease’s natural history [18, 19].

The rationale for optic canal decompression in NAION is grounded in the compartment syndrome model of the optic nerve head. Our findings support the ‘osseous compartment syndrome’ hypothesis. By alleviating the mechanical constraint of the rigid optic canal, ETOCD may break the cycle of compression and secondary ischemic injury, potentially restoring local perfusion and interrupting the ischemic cascade [20].

Specifically, our surgical approach underscores the necessity of addressing the compartment syndrome at both the osseous and fascial levels. While the removal of the canal wall alleviates the macro-structural constraint of the rigid bone, the subsequent sheath fenestration (via punctate incisions) serves as a targeted pressure release for the swollen axons themselves. This dual-action mechanism is hypothesized to be crucial for restoring microcirculatory perfusion; by addressing both the extrinsic bony boundaries and the intrinsic fascial restriction, we propose that this integrated approach targets the self-perpetuating vicious cycle of NAION pathophysiology at multiple structural levels.

The identification of the 150 μm threshold likely represents the tipping point between ‘reversible axoplasmic stasis’ and ‘irreversible compartmental tamponade’. Furthermore, our qualitative OCTA findings suggest this hypothesis by providing visual illustrations that decompression within this window may facilitate the restoration of microcirculatory perfusion, potentially interrupting the vicious cycle. While the primary pathology in NAION is microvascular rather than traumatic, the principle of relieving structural compression remains applicable, especially in patients with anatomical risk factors such as a small or crowded optic disc [21].

This mechanistic perspective is supported by evidence of altered ocular hemodynamics in NAION. Laser speckle flowgraphy studies have demonstrated distinct perfusion deficits in the optic nerve head and peripapillary choroid in ischemic optic neuropathies [22]. Consistently, OCT angiography has revealed reduced peripapillary vessel density in affected eyes compared to fellow eyes and healthy controls, underscoring the vascular basis of the disease [23–26]. In our cohort, postoperative OCTA parameters in the ETOCD group showed no significant decrease in disc and peripapillary vessel density, which may indicate a stabilizing effect of decompression on the microvasculature and a potential halt in the progressive vascular rarefaction typical of NAION’s natural course [27, 28].

A key finding of this study is the differential treatment response according to baseline RNFL thickness. The significant interaction between treatment effect and baseline RNFL—particularly the pronounced benefit of ETOCD in patients with moderate edema (< 150 μm, OR = 7.88)—suggests a structural therapeutic window. In these patients, axons may be compromised but still viable, and thus salvageable through decompression. Conversely, massive edema (≥ 150 μm) may indicate extensive, irreversible infarction, where mechanical decompression is unlikely to restore function. The observed RNFL thinning over time in both groups aligns with the natural history of NAION, wherein initial edema resolves into atrophy [29, 30]. Nevertheless, the superior functional outcomes in the ETOCD group despite similar structural thinning imply that decompression may confer neuroprotective effects beyond what RNFL thickness alone reflects—possibly through improved venous drainage, edema reduction, and mitigation of secondary inflammatory injury.

Complementing the structural findings, our analysis of disease duration revealed a widely extended therapeutic window [31, 32]. Contrary to the IONDT’s strict 14-day cutoff, we found no significant interaction between ‘time from onset’ and surgical efficacy (P > 0.05). ETOCD conferred visual benefits even in patients treated during the subacute to early atrophic phases (median 60 days). This implies that relieving the bony constraint may rescue ‘idling’ axons that persist beyond the resolution of acute edema.

Multivariable regression supported the independent predictive value of ETOCD, with an adjusted coefficient of − 0.41 for postoperative BCVA improvement, indicating a substantial treatment effect after accounting for confounders. Preoperative BCVA consistently emerged as a strong positive predictor in both univariable and multivariable models, underscoring the prognostic relevance of baseline visual function. Notably, the shift from nonsignificance in the unadjusted analysis to strong significance after adjustment suggests that key confounding variables were adequately controlled, thereby strengthening the validity of our conclusions regarding ETOCD efficacy.

Historically, optic canal decompression was performed via open transcranial or external approaches, which carried greater morbidity [33]. Advances in endoscopic and image-guided techniques have established ETOCD as a minimally invasive and precise alternative. Its efficacy has been demonstrated in other compressive optic neuropathies, including traumatic optic neuropathy—even in preschool-aged children—and benign fibro-osseous lesions involving the canal, where visual improvement has been reported in a substantial proportion of cases [34, 35].

Within the current therapeutic landscape for NAION—where no intervention has consistently demonstrated efficacy in controlled studies—the visual improvement and responder rate observed with ETOCD (63.3% vs. 31.7% with medical management) appear noteworthy. Systematic reviews have highlighted the limited and inconsistent benefits of existing pharmacologic options, including corticosteroids, anti-VEGF agents, and neuroprotective drugs [18, 19, 36]. For instance, early intravitreal triamcinolone has been associated with improvement rates of 49–55% [12, 37], and intravitreal bevacizumab has shown some effect on disease progression in a case-control setting [11]. The mechanism of ETOCD differs fundamentally from these pharmacologic strategies: rather than targeting edema, vascular permeability, or neuronal survival indirectly, it directly alleviates the structural constraint within the optic canal, addressing a proposed “bony compartment” component of NAION pathophysiology.

Regarding safety, ETOCD carries specific risks inherent to skull-base surgery, such as potential injury to the internal carotid artery or cerebrospinal fluid leak, which demand considerable surgical expertise [38]. Although no major complications occurred in our series, reported cases of internal carotid pseudoaneurysm during ETOCD underscore the need for careful patient selection and procedural experience [38]. By comparison, medical interventions are not without risk: intravitreal injections may lead to endophthalmitis, ocular hypertension, or retinal detachment, while systemic corticosteroids can worsen common comorbidities such as hypertension and diabetes. Thus, the risk–benefit profile of ETOCD must be individualized, weighing anatomical factors, systemic health, and disease severity against the potential for visual recovery.

Several limitations of this study should be acknowledged. First, the non-randomized, retrospective design may introduce selection bias. Notably, standardized neuroimaging (e.g., MRI) was not uniformly performed due to the retrospective design. While diagnoses were verified through longitudinal follow-up, the reliance on clinical criteria may introduce potential diagnostic bias. Additionally, although baseline characteristics were largely comparable between groups, patients in the surgical cohort presented with worse initial vision (median BCVA 1.20 vs. 0.80 logMAR), raising the possibility that regression toward the mean contributed to the observed improvement. Nevertheless, the magnitude of the treatment effect (OR = 2.76) suggests a benefit beyond statistical artifact. Second, despite the use of multivariate adjustment and subgroup analyses, unmeasured confounders may persist. Third, given the exploratory nature of this study, no formal correction for multiple comparisons (e.g., Bonferroni) was applied; the risk of spurious findings due to multiple testing cannot be excluded, and our results require prospective validation. Fourth, although the sample size was adequate for detecting primary outcome differences, it may have been underpowered for certain subgroup comparisons and safety assessments. Future prospective studies with larger cohorts, longer follow-up, and ideally randomized allocation are needed to confirm these findings and evaluate the long-term efficacy and safety of ETOCD in NAION.

Beyond the immediate outcomes, this study highlights the potential role of baseline retinal nerve fiber layer thickness as a treatment-effect modifier. If validated, preoperative RNFL assessment could advance toward a predictive biomarker, assisting in the selection of patients most likely to benefit from surgical decompression. Such an approach aligns with the growing emphasis on personalized medicine in neuro-ophthalmology, where objective structural and vascular parameters are increasingly used to guide therapeutic decisions [29, 39]. Specifically, the 150 μm RNFL threshold could serve as a valuable enrichment criterion for future prospective randomized controlled trials. By selecting patients most likely to respond to surgical intervention, this biomarker-driven strategy could enhance the statistical power of such studies and facilitate the development of standardized management protocols.

Several key questions remain to be addressed in future research. The optimal timing for ETOCD intervention requires clarification; while our study included patients treated within a defined period, earlier surgery—potentially in the hyperacute phase—might yield greater benefit. Furthermore, the precise mechanisms underlying ETOCD’s therapeutic effect—whether predominantly mediated by mechanical decompression, improved local hemodynamics, or modulation of secondary inflammatory pathways—warrant deeper investigation. Advanced vascular imaging, including serial OCT angiography and Doppler flowmetry, could objectively quantify perioperative changes in ocular perfusion and help elucidate these mechanisms [40, 41]. Additionally, exploring combination strategies that integrate surgical decompression with pharmacologic agents targeting complementary pathophysiologic pathways represents a promising future direction.

In summary, our findings indicate that endoscopic transnasal optic canal decompression may offer a valuable therapeutic alternative for patients with NAION, especially those presenting with moderate optic disc edema. These results underscore the need for a prospective, randomized controlled trial to rigorously evaluate the efficacy, safety, and optimal application of osseous decompression in the management of this vision-threatening condition.

## Data Availability

The datasets used and/or analyzed during the current study are available from the corresponding author on reasonable request.

## Abbreviations

NAION: Nonarteritic anterior ischemic optic neuropathy
ETOCD: Endoscopic transnasal optic canal decompression
BCVA: Best-corrected visual acuity
RNFL: Retinal nerve fiber layer
OCT: Optical coherence tomography
OCTA: Optical coherence tomography angiography
IONDT: Ischemic Optic Neuropathy Decompression Trial
VD: Vessel density
RPC: Radial peripapillary capillaries
VF: Visual field
MD: Mean deviation
PSD: Pattern standard deviation
CI: Confidence interval
OR: Odds ratio
LogMAR: Logarithm of the minimum angle of resolution

## References

[1] Hattenhauer MG, Leavitt JA, Hodge DO, Grill R, Gray DT. Incidence of nonarteritic anterior ischemic optic neuropathy. Am J Ophthalmol. 1997;123(1):103–7. 10.1016/s0002-9394(14)70999-7. 9186104 10.1016/s0002-9394(14)70999-7

[2] Morrow MJ. Ischemic optic neuropathy. 2019. 10.1212/CON.0000000000000767 31584535

[3] Parthasarathi P, Moss HE. Review of evidence for treatments of acute non arteritic anterior ischemic optic neuropathy. Eye (Lond). 2024;38(12):2267–78. 10.1038/s41433-024-03136-8. 38778140 10.1038/s41433-024-03136-8 PMC11306228

[4] Verriello L, Pauletto G, Zeppieri M, Lorenzut S, Bertolotti C, Gagliano C, et al. Therapeutic approaches for toxic optic neuropathies: Insights from methanol-induced optic neuropathy and NAION treatments. Diagnostics (Basel). 2025;15(22):2883. 10.3390/diagnostics15222883. 41300907 10.3390/diagnostics15222883 PMC12650965

[5] Wang L, Volkow ND, Kaelber DC, Xu R. Semaglutide or tirzepatide and optic nerve and visual pathway disorders in type 2 diabetes. JAMA Netw Open. 2025;8(8):e2526327. 10.1001/jamanetworkopen.2025.26327. 40788646 10.1001/jamanetworkopen.2025.26327 PMC12340659

[6] Fard MA, Ghahvechian H, Sahrayan A, Subramanian PS. Early macular vessel density loss in acute ischemic optic neuropathy compared to papilledema: Implications for pathogenesis. Transl Vis Sci Technol. 2018;7(5):10. 10.1167/tvst.7.5.10. 30271677 10.1167/tvst.7.5.10 PMC6159734

[7] Nagia L, Huisingh C, Johnstone J, Kline LB, Clark M, Girard MJA, et al. Peripapillary pachychoroid in nonarteritic anterior ischemic optic neuropathy. Invest Ophthalmol Vis Sci. 2016;57(11):4679–85. 10.1167/iovs.16-19315. 27583829 10.1167/iovs.16-19315 PMC5017268

[8] Evaluation. and management of nonarteritic anterior ischemic optic neuropathy: a national survey | graefe’s archive for clinical and experimental ophthalmology. https://link.springer.com/article/10.1007/s00417-024-06512-y. Accessed 10 Dec 2025. 10.1007/s00417-024-06512-y PMC11458737 38748211

[9] Chen T, Song D, Shan G, Wang K, Wang Y, Ma J, et al. The association between diabetes mellitus and nonarteritic anterior ischemic optic neuropathy: A systematic review and meta-analysis. PLoS ONE. 2013;8(9):e76653. 10.1371/journal.pone.0076653. 24098798 10.1371/journal.pone.0076653 PMC3786911

[10] Levin LA, Bhatti MT, Klier S, Morgenstern R, Szanto D, Miller NR, et al. A randomized sham-controlled phase 2/3 trial of QPI-1007 for acute nonarteritic anterior ischemic optic neuropathy. Ophthalmology. 2025. 10.1016/j.ophtha.2025.07.039. 40816607 10.1016/j.ophtha.2025.07.039

[11] Najafi A, Mesri R, Ojaghi H, Iranpour S. The beneficial effects of bevacizumab on the progression of non-arteritic anterior ischemic optic neuropathy: A case-control study. Annals Med Surg. 2025;87(10):6272. 10.1097/MS9.0000000000003484. 10.1097/MS9.0000000000003484 PMC12578095 41181441

[12] Radoi C, Garcia T, Brugniart C, Ducasse A, Arndt C. Intravitreal triamcinolone injections in non-arteritic anterior ischemic optic neuropathy. Graefes Arch Clin Exp Ophthalmol. 2014;252(2):339–45. 10.1007/s00417-013-2499-9. 24178807 10.1007/s00417-013-2499-9

[13] Dickersin K. Optic nerve decompression surgery for nonarteritic anterior ischemic optic neuropathy (NAION) is not effective and may be harmful. JAMA: J Am Med Association. 1995;273(8):625. 10.1001/jama.1995.03520320035038. 7844872

[14] He M, Yang Z, Shi Z, Chen Y, Zhao X. Clinical features, management, and prognosis factors of traumatic optic neuropathy. Surv Ophthalmol. 2026;71(1):168–78. 10.1016/j.survophthal.2025.07.012. 40749877 10.1016/j.survophthal.2025.07.012

[15] Tu Y, Yan W, Hu X, Wu W, Ye J. Retinal vasculature in indirect traumatic optic neuropathy with endoscopic optic canal decompression. J Transl Med. 2025;23(1):928. 10.1186/s12967-025-06968-4. 40826102 10.1186/s12967-025-06968-4 PMC12363014

[16] Zhang W, Komatsu F, Kato Y. How i do it: Endoscopic transposition technique for hemifacial spasm caused by AICA compression. Acta Neurochir (Wien). 2025. 10.1007/s00701-025-06723-0. 41339533 10.1007/s00701-025-06723-0 PMC12695956

[17] Wu J, Zheng W, Fang L, Yun Z, Zhang C, Li C. Innovative design and accuracy of optical and electromagnetic integrated surgical navigation system: Phantom and in-vivo studies. Int J Surg. 2025. 10.1097/JS9.0000000000004117. 41346264 10.1097/JS9.0000000000004117

[18] Lantos K, Dömötör ZR, Farkas N, Kiss S, Szakács Z, Garami A, et al. Efficacy of treatments in nonarteritic ischemic optic neuropathy: A systematic review and meta-analysis. Int J Environ Res Public Health. 2022;19(5):2718. 10.3390/ijerph19052718. 35270411 10.3390/ijerph19052718 PMC8910678

[19] Badla O, Badla BA, Almobayed A, Mendoza C, Kishor K, Bhattacharya SK. Ischemic optic neuropathy: A review of current and potential future pharmacotherapies. Pharmaceuticals (Basel). 2024;17(10):1281. 10.3390/ph17101281. 39458922 10.3390/ph17101281 PMC11510045

[20] Biousse V, Newman NJ. Ischemic optic neuropathies. N Engl J Med. 2015;372(25):2428–36. 10.1056/NEJMra1413352. 26083207 10.1056/NEJMra1413352

[21] Ahmadi H, Hamann S. Anterior ischemic optic neuropathy in patients treated with semaglutide: Report of four cases with a possible association. BMC Ophthalmol. 2025;25(1):132. 10.1186/s12886-025-03958-4. 40087651 10.1186/s12886-025-03958-4 PMC11908077

[22] Yamaguchi C, Kiyota N, Himori N, Omodaka K, Tsuda S, Nakazawa T. Differentiating optic neuropathies using laser speckle flowgraphy: Evaluating blood flow patterns in the optic nerve head and peripapillary choroid. Acta Ophthalmol. 2025;103(1):e49–57. 10.1111/aos.16747. 39136108 10.1111/aos.16747 PMC11704823

[23] Pugazhendhi S, Yu M, Zhou G, Chen Y, Wang R, Liao YJ. Peripapillary and macular microvasculature features of non-arteritic anterior ischemic optic neuropathy. Front Med (Lausanne). 2022;9:1033838. 10.3389/fmed.2022.1033838. 36714135 10.3389/fmed.2022.1033838 PMC9877420

[24] Hondur G, Budakoglu O. Peripapillary microvascular and structural parameters in atrophic nonarteritic anterior ischemic optic neuropathy and their unaffected fellow eyes. J Neuroophthalmol. 2022;42(4):489–94. 10.1097/WNO.0000000000001542. 35421878 10.1097/WNO.0000000000001542

[25] Su Y, Zhang S, Zhang G, Liu Y, Du Z, Li D, et al. Quantification of peripapillary vessel density in non-arteritic anterior ischemic optic neuropathy patients with optical coherence tomography angiography. Quant Imaging Med Surg. 2022;12(2):1549–57. 10.21037/qims-21-800. 35111647 10.21037/qims-21-800 PMC8739086

[26] Ling L, Ji K, Xie L, Gao F, Zhang Q, Xing Y, et al. Optical coherence tomography angiography assessment of the peripapillary vessel density and structure in patients with nonarteritic anterior ischemic optic neuropathy: A meta-analysis. Biomed Res Int. 2020;2020:1359120. 10.1155/2020/1359120. 33178816 10.1155/2020/1359120 PMC7647740

[27] Xiao Q, Sun C-B. Detecting changes in the blood flow of the optic disk in patients with nonarteritic anterior ischemic optic neuropathy via optical coherence tomography-angiography. Front Neurol. 2023;14:1140770. 10.3389/fneur.2023.1140770. 37034068 10.3389/fneur.2023.1140770 PMC10081673

[28] Said OM, Saif AT, Owis E, Shaheen MHAEG. Peripapillary and macular vascular changes in unilateral nonarteritic anterior ischemic optic neuropathy: An optical coherence tomography angiography study. Indian J Ophthalmol. 2025;73(11):1633–9. 10.4103/IJO.IJO_1692_24. 41148018 10.4103/IJO.IJO_1692_24 PMC12659851

[29] Razaghi G, Hedayati E, Hejazi M, Kafieh R, Samadi M, Ritch R, et al. Measurement of retinal nerve fiber layer thickness with a deep learning algorithm in ischemic optic neuropathy and optic neuritis. Sci Rep. 2022;12(1):17109. 10.1038/s41598-022-22135-x. 36224300 10.1038/s41598-022-22135-x PMC9556618

[30] Akbulut S, Pekel G, Pekel E, Cetin EN. Alterations in retinal and choroidal thickness following nonarteritic anterior ischemic optic neuropathy. Int Ophthalmol. 2021;41(8):2723–8. 10.1007/s10792-021-01829-7. 33818675 10.1007/s10792-021-01829-7

[31] Atkins EJ, Bruce BB, Newman NJ, Biousse V. Treatment of nonarteritic anterior ischemic optic neuropathy. Surv Ophthalmol. 2010;55(1):47–63. 10.1016/j.survophthal.2009.06.008. 20006051 10.1016/j.survophthal.2009.06.008 PMC3721361

[32] Anterior ischemic optic neuropathy. Part 2. A discussion for physicians - EyeRounds. https://webeye.ophth.uiowa.edu/eyeforum/article/AION/AION-part2.htm. Accessed 7 Jan 2026.

[33] Beseoglu K, Lodes S, Stummer W, Steiger H-J, Hänggi D. The transorbital keyhole approach: Early and long-term outcome analysis of approach-related morbidity and cosmetic results. Technical note. J Neurosurg. 2011;114(3):852–6. 10.3171/2010.9.JNS1095. 21029037 10.3171/2010.9.JNS1095

[34] Thakar A, Mahapatra AK, Tandon DA. Delayed optic nerve decompression for indirect optic nerve injury. Laryngoscope. 2003;113(1):112–9. 10.1097/00005537-200301000-00021. 12514393 10.1097/00005537-200301000-00021

[35] Behbahani M, Fernando S, Peng S, Fernandez LG, Hajnas N, Sharma S, et al. Endoscopic endonasal optic nerve decompression: Treatment of fibrous dysplasia in a pediatric population. J Neurosurg Pediatr. 2023;31(2):179–85. 10.3171/2022.9.PEDS22313. 36401542 10.3171/2022.9.PEDS22313

[36] Budihardja BM, Anggraini E, Pratiwi RW, Nastiti AD, Nusanti S. Neuroprotective strategies for nonarteritic anterior ischemic optic neuropathy: A systematic review. Korean J Ophthalmol. 2023;37(4):328–39. 10.3341/kjo.2022.0166. 37563973 10.3341/kjo.2022.0166 PMC10427903

[37] Durbant E, Radoi C, Garcia T, Denoyer A, Arndt C. Intravitreal triamcinolone injections in non-arteritic anterior ischemic optic neuropathy - a retrospective report. J Fr Ophtalmol. 2021;44(6):777–85. 10.1016/j.jfo.2020.07.027. 34053770 10.1016/j.jfo.2020.07.027

[38] Yu Z, Qi J, Wang L, Yang X, Liu Z, Chen X, et al. Managing intraoperative rupture of internal carotid pseudoaneurysms during endoscopic transnasal optic canal decompression: A case report. Front Neurol. 2024;15:1382793. 10.3389/fneur.2024.1382793. 38962479 10.3389/fneur.2024.1382793 PMC11220115

[39] Jalili J, Nadimi M, Jafari B, Esfandiari A, Mojarad M, Subramanian PS, et al. Vessel density features of optical coherence tomography angiography for classification of optic neuropathies using machine learning. J Neuroophthalmol. 2024;44(1):41–6. 10.1097/WNO.0000000000001925. 37440373 10.1097/WNO.0000000000001925

[40] Fu Z, Li H, Wang Y. Implication of retrobulbar and internal carotid artery blood-flow-volume alterations for the pathogenesis of non-arteritic anterior ischemic optic neuropathy. BMC Ophthalmol. 2021;21(1):309. 10.1186/s12886-021-02075-2. 34433431 10.1186/s12886-021-02075-2 PMC8390251

[41] Augstburger E, Ballino A, Keilani C, Robin M, Baudouin C, Labbé A. Follow-up of nonarteritic anterior ischemic optic neuropathy with optical coherence tomography angiography. Invest Ophthalmol Vis Sci. 2021;62(2):42. 10.1167/iovs.62.4.42. 33635311 10.1167/iovs.62.4.42 PMC7945964

